# Pancreatitis‐Related Encapsulated Fluid Collections Associated With a Pancreatic Duct Stent Retained for 13 Years in Chronic Alcoholic Pancreatitis: A Case Report

**DOI:** 10.1002/deo2.70427

**Published:** 2026-09-10

**Authors:** Koichi Soga, Ou Takagi, Yutarou Koike, Kohei Inaba, Mayumi Yamaguchi, Yugo Tetsuka, Masaru Kuwada, Daiki Nemoto, Ikuhiro Kobori, Katsutoshi Sugimoto

**Affiliations:** ^1^ Department of Gastroenterology Dokkyo Medical University Saitama Medical Center Saitama Japan

**Keywords:** chronic pancreatitis, pancreatic duct drainage, pancreatic duct stent, pancreatic fluid collection, retained stent

## Abstract

Pancreatic duct stenting is an established treatment for ductal decompression in obstructive chronic pancreatitis. However, very long‐term stent retention may be associated with late complications, particularly in patients with advanced chronic pancreatitis. A 65‐year‐old man with chronic alcoholic pancreatitis underwent pancreatic duct stenting for a main pancreatic duct stricture, but follow‐up was discontinued, and the stent remained in situ for approximately 13 years. He presented with fever and loss of appetite. Computed tomography showed diffuse pancreatic calcifications and pancreatitis‐related encapsulated peripancreatic and pelvic fluid collections. The pelvic collection appeared relatively homogeneous, suggesting a pancreatic fluid leak‐associated or pseudocyst‐like collection rather than definitive walled‐off necrosis. After removal of the retained stent, a new pancreatic duct plastic stent was placed. The collections improved after conservative treatment and stent exchange. This case highlights the need for strict surveillance after pancreatic duct stenting.

## 1 Introduction

Chronic alcoholic pancreatitis is characterized by pancreatic calcification, pancreatic stones, main pancreatic duct stricture, ductal dilatation, and parenchymal atrophy. These changes increase resistance to pancreatic juice outflow and may promote recurrent pain, ductal hypertension, and acute inflammatory exacerbation. Endoscopic pancreatic duct stenting is performed to decompress the main pancreatic duct in obstructive chronic pancreatitis; however, pancreatic duct stents require scheduled surveillance and exchange because stent occlusion, migration, fracture, stent‐induced ductal changes, pancreatitis, and infection may occur [1].

When pancreatic duct drainage is impaired, pancreatitis‐related fluid collections may develop, including pancreatic fluid leak‐associated collections resulting from pancreatic duct disruption [2], pseudocyst‐like collections, and walled‐off necrosis (WON) in necrotizing pancreatitis [3, 4]. Reports of pancreatic duct stents retained for >10 years are rare [5, 6]. We report a case of chronic alcoholic pancreatitis in which a pancreatic duct stent retained for approximately 13 years was associated with encapsulated peripancreatic and pelvic fluid collections.

## 2 Case Report

A 65‐year‐old man with alcohol dependence and chronic alcoholic pancreatitis had previously undergone endoscopic pancreatic duct stenting for a main pancreatic duct stricture. Scheduled stent exchange was initially performed, but follow‐up was discontinued, and the stent remained in situ for approximately 13 years. Alcohol consumption continued during the follow‐up interruption, although the exact daily amount was unclear.

**FIGURE 1:**
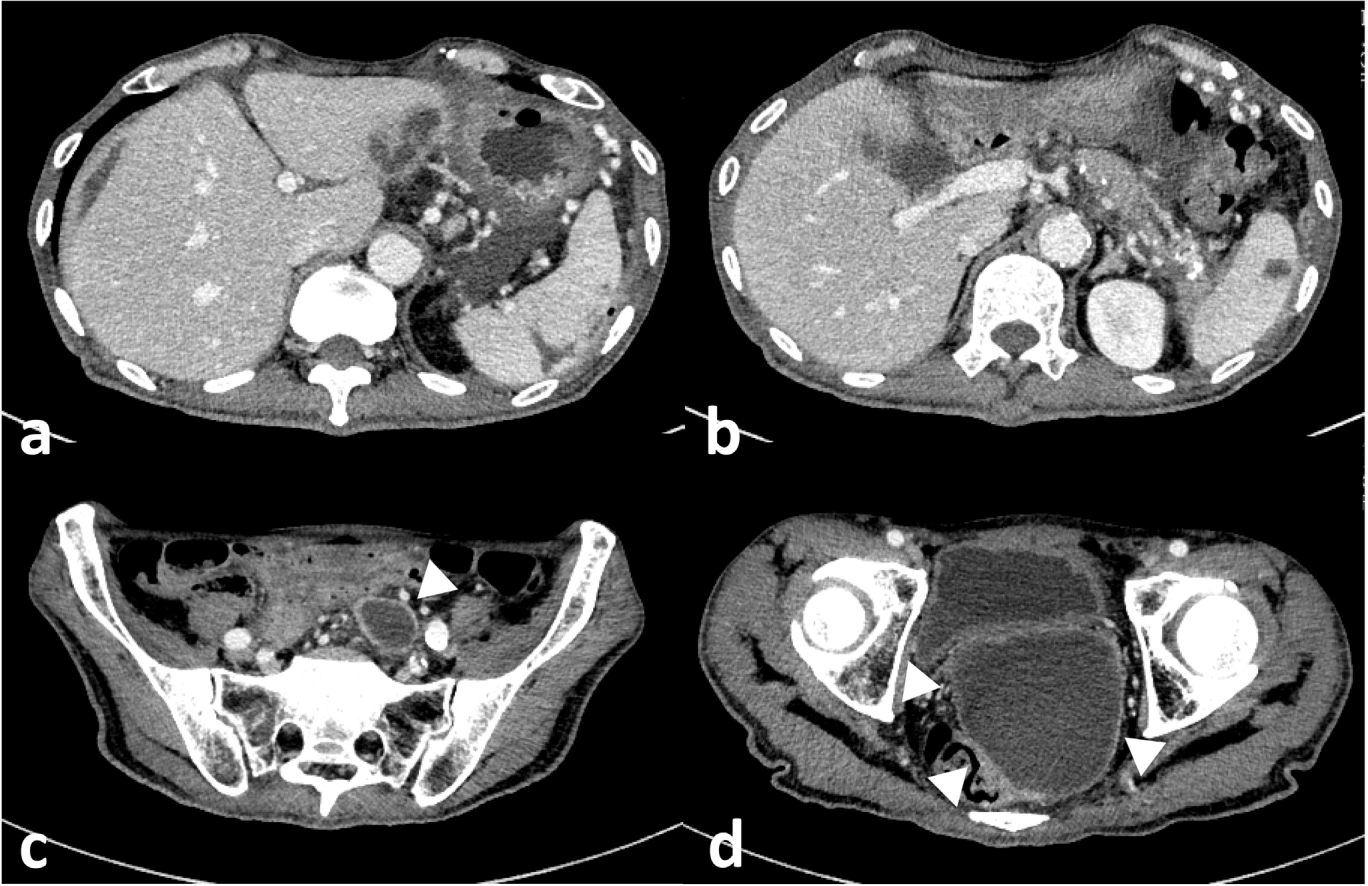
Contrast‐enhanced computed tomography findings at the onset of acute exacerbation of chronic pancreatitis. (a, b) Contrast‐enhanced computed tomography showing inflammatory changes around the pancreas with multiple pancreatic calcifications, consistent with chronic pancreatitis. Peripancreatic fluid collections are observed around the pancreatic body and tail. (c, d) Encapsulated pelvic fluid collections (arrowheads) are visible adjacent to the rectosigmoid colon and urinary bladder. These findings were considered compatible with pancreatitis‐related encapsulated peripancreatic and pelvic fluid collections. Because obvious necrotic debris was not clearly identified and the pelvic collection appeared relatively homogeneous, a pancreatic fluid leak‐associated or pseudocyst‐like collection was also considered.

He later presented to a local hospital with fever and loss of appetite. Laboratory tests showed marked systemic inflammation: white blood cell count, 13,140/µL; C‐reactive protein level, 21.73 mg/dL (Table S1). Contrast‐enhanced computed tomography (CT) revealed diffuse pancreatic calcifications and encapsulated peripancreatic fluid collections around the pancreatic body and tail. Although WON was initially suspected, no obvious necrotic debris was identified. Follow‐up abdominal CT showed progression of an encapsulated fluid collection extending into the pelvic cavity despite initial conservative treatment. It appeared relatively homogeneous, suggesting a pancreatic fluid leak‐associated or pseudocyst‐like collection rather than a definite necrotic collection (Figure 1). The patient was referred to our institution for possible transrectal drainage and management of the retained pancreatic duct stent.

Endoscopic retrograde cholangiopancreatography was performed. Duodenoscopy showed the retained pancreatic duct stent protruding from the major papilla, with inflammatory polypoid mucosal changes around the papillary orifice. Preprocedural abdominal radiography showed the retained stent with adjacent pancreatic stones. Because the papillary orifice was deformed and narrowed, pancreatic sphincterotomy was performed before stent extraction to reduce extraction resistance and secure repeated device access in case of unexpected stent fracture, material deterioration, or bleeding. The sphincterotome was inserted into the main pancreatic duct alongside the retained stent. The retained stent was grasped using a snare catheter and withdrawn under endoscopic and fluoroscopic guidance. Because the stent could not be safely pulled into the working channel, the grasped stent and endoscope were withdrawn together while maintaining coaxial alignment. Gentle traction was applied under direct visualization. The procedure was discontinued if strong resistance was encountered. The stent was removed intact, and no perforation or clinically significant bleeding occurred thereafter.

**FIGURE 2:**
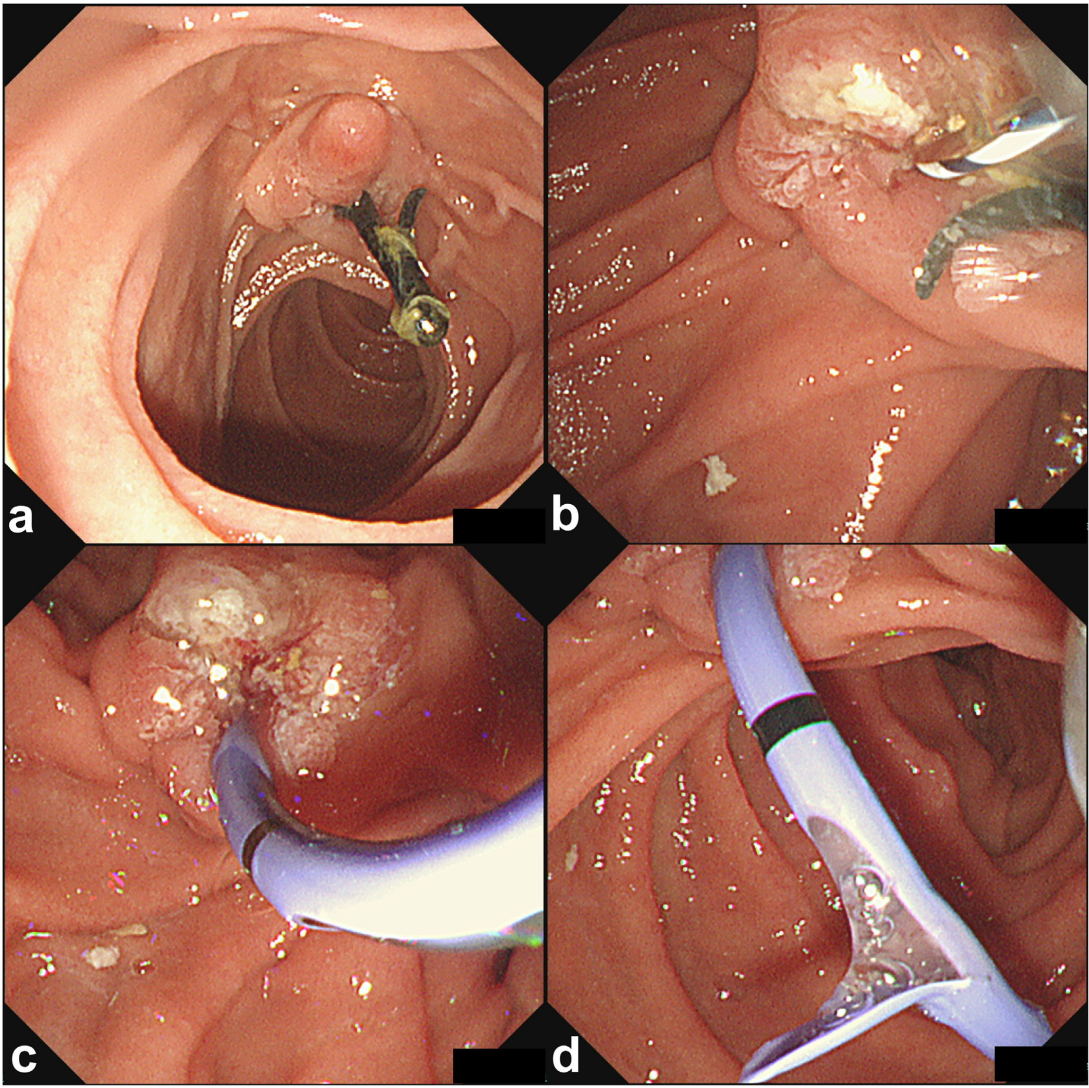
Endoscopic findings and pancreatic duct stent exchange in a patient with chronic alcoholic pancreatitis. (a) Duodenoscopic image showing a previously placed pancreatic duct stent protruding from the major papilla. Inflammatory polypoid mucosal changes are observed around the papillary orifice, suggesting possible chronic mechanical irritation associated with the long‐term retained pancreatic duct stent. Pancreatic sphincterotomy was performed before stent extraction to reduce resistance at the deformed papillary orifice. (b) Endoscopic manipulation was performed around the papilla, where whitish debris and inflammatory mucosal changes are shown adjacent to the retained pancreatic duct stent. (c) Removal of the retained pancreatic duct stent was attempted endoscopically. The procedure was technically challenging because of papillary deformity and limited maneuverability of the long‐retained stent. (d) After removal of the retained stent and pancreatography‐guided evaluation, a new pancreatic duct plastic stent was successfully placed to maintain pancreatic duct drainage.

**FIGURE 3:**
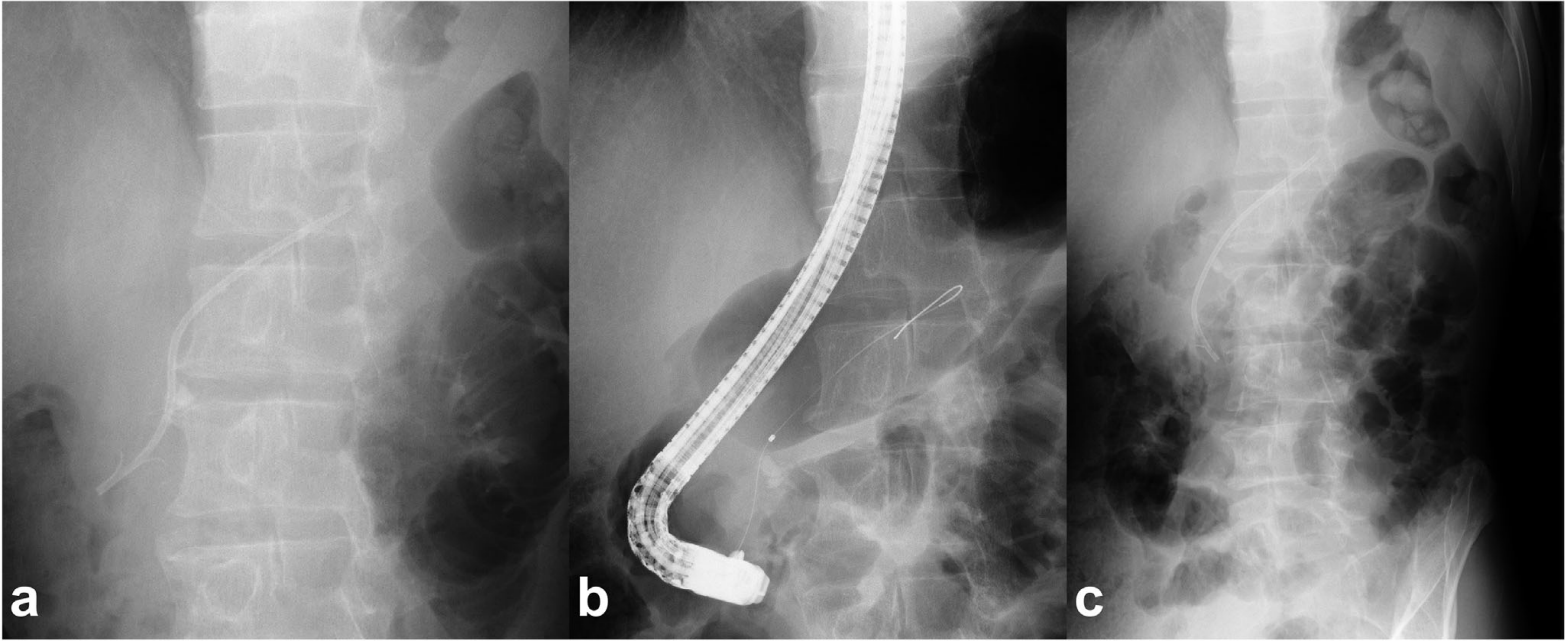
Radiographic and fluoroscopic findings during exchange of a pancreatic duct stent retained for 13 years. (a) Radiographic imaging showing the long‐term indwelling pancreatic duct stent that had been retained for 13 years. A pancreatic stone is shown adjacent to the retained stent. (b) A guidewire was inserted into the pancreatic duct, and endoscopic removal of the retained pancreatic duct stent was performed. No firm adhesion between the stent and the pancreatic stone was observed, and there was no evidence of stent fragmentation due to material deterioration during removal. (c) After removal of the retained stent, a new 7‐Fr 10‐cm pancreatic plastic stent was placed across the pancreatic duct stricture. Although stent insertion was technically challenging because of the pancreatic stone and ductal narrowing, successful stent placement was achieved.

Pancreatography revealed a main pancreatic duct stricture in the pancreatic head corresponding to the stone‐bearing segment. A new 7‐Fr, 10‐cm pancreatic plastic stent was successfully placed to maintain pancreatic duct drainage (Figures 2 and 3; Video S1).

**FIGURE 4:**
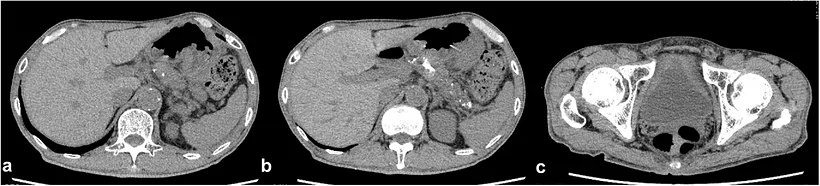
Follow‐up computed tomography after conservative management and pancreatic duct stent exchange. (a, b) Follow‐up computed tomography showing persistent diffuse pancreatic calcifications, consistent with advanced chronic pancreatitis. However, the peripancreatic fluid collections observed at the initial presentation had markedly decreased. (c) The pelvic encapsulated fluid collection had nearly disappeared. These findings suggested substantial improvement of pancreatitis‐related encapsulated peripancreatic and pelvic fluid collections after conservative treatment and maintenance of pancreatic duct drainage by stent exchange.

Follow‐up CT 3 months later showed a marked reduction in the peripancreatic collections and near‐complete disappearance of the pelvic collection; therefore, transrectal drainage was not performed (Figure 4). Scheduled pancreatic duct stent exchange was continued approximately every 3 months. The collections did not recur. Approximately 12 months later, an 8.5‐Fr, 10‐cm pancreatic duct stent was placed, and the patient was referred for subsequent extracorporeal shock wave lithotripsy (ESWL) for obstructive pancreatic stones. Although the pancreatic stones impacted within the branch ducts could not be completely fragmented or retrieved, successful stone fragmentation and clearance within the main pancreatic duct resulted in satisfactory pancreatic duct decompression. At 4 months after ESWL, the patient was clinically stable without recurrence of the peripancreatic or pelvic collections, and the pancreatic duct stent was removed.

## 3 Discussion

This case is noteworthy because a pancreatic duct stent retained for approximately 13 years was associated with acute exacerbation of chronic alcoholic pancreatitis and pancreatitis‐related encapsulated peripancreatic and pelvic fluid collections. The major clinical message is not that the retained stent itself was the primary cause but that inadequate follow‐up after pancreatic duct stenting may permit progressive impairment of pancreatic duct drainage to develop silently in the presence of pancreatic stones, main pancreatic duct stricture, and continued alcohol consumption.

Diagnosis of peripancreatic and pelvic collections requires careful interpretation. Although the peripancreatic collection was initially considered compatible with suspected WON because it developed after acute inflammatory exacerbation and showed encapsulation, obvious solid necrotic debris was not clearly identified. Additionally, the pelvic collection appeared relatively homogeneous. Therefore, alternative diagnoses, including pancreatic fluid leak‐associated collection, pseudocyst‐like collection, and disconnected pancreatic duct syndrome, should be considered [2]. In our case, pancreatic fluid leak or a pseudocyst‐like collection was plausible because the patient had pancreatic stones, a main pancreatic duct stricture, and improvement after pancreatic duct stent exchange. However, fluid analysis was unavailable because direct drainage was not performed. Disconnected pancreatic duct syndrome was also considered, but complete ductal disruption was not demonstrated, and no recurrence occurred during scheduled stent exchange or up to the time of stent removal. Therefore, we interpreted the lesions as pancreatitis‐related encapsulated fluid collections rather than definitive WON.

Several mechanisms may explain this clinical course. First, prolonged stent retention may have contributed to chronic local mechanical irritation and ductal impairment. Duodenoscopy showed papillary deformity and inflammatory polypoid mucosal changes around the papillary orifice. Histological evaluation was not performed; therefore, these changes were described based on endoscopic findings, and definitive histopathological interpretation was avoided. Previous studies have shown that pancreatic duct stenting can induce ductal changes in chronic pancreatitis, including stricture‐like changes, and that some may persist or become irreversible with continued plastic stent placement [7, 8]. Second, advanced chronic alcoholic pancreatitis with pancreatic stones and main pancreatic duct stricture likely increased resistance to pancreatic juice outflow. Thus, the clinical course was likely multifactorial rather than solely attributable to the retained stent. Because direct evidence of luminal occlusion was unavailable, stent dysfunction remains presumptive. Pancreatic stones and main pancreatic duct stricture may have been major contributors to impaired pancreatic drainage. Even after retained stent removal, the stone‐bearing ductal segment remained difficult to traverse, supporting a possible vicious cycle of ductal narrowing, impaired outflow, ductal hypertension, and inflammation, in which the retained stent may have contributed but was not proven to be the sole driver. Third, pancreatitis‐related collections can extend from the peripancreatic region along retroperitoneal spaces and paracolic gutters into the pelvis. Pelvic extension of WON treated via a transrectal approach has been described [9]. In this case, the pelvic collection was interpreted as a remote pancreatitis‐related collection rather than an isolated pelvic abscess.

The collections improved after conservative management and pancreatic duct stent exchange without transrectal drainage, suggesting that restored ductal drainage might have reduced intraductal pressure and ongoing pancreatic juice leakage or inflammation. Subsequent ESWL achieved fragmentation and clearance of the main pancreatic duct stones, permitting stent removal after 4 months. Withdrawal of a retained pancreatic duct stent together with the endoscope is not routine because of stent fracture and mucosal injury risks. In this case, guidewire access was secured, the stent was mobilized without marked resistance, and gentle coaxial traction was applied under endoscopic and fluoroscopic guidance; the retrieved stent remained intact.

Our findings expand the literature on stents retained for >10 years [5, 6] and suggest that, in advanced chronic pancreatitis, a forgotten stent may contribute to impaired pancreatic drainage as part of a multifactorial process. Patients with retained stents who develop pancreatitis‐related collections should be evaluated for stent dysfunction, ductal stricture, and pancreatic stones, with definitive stone treatment considered when appropriate [1].

In conclusion, pancreatitis‐related encapsulated peripancreatic and pelvic fluid collections occurred in a patient with advanced chronic alcoholic pancreatitis and a pancreatic duct stent retained for 13 years. Long‐term stent retention may have contributed to impaired pancreatic drainage within a multifactorial process, underscoring the importance of strict surveillance and active recall after pancreatic duct stenting.

## Author Contributions

**Koichi Soga**: conceptualization, data curation, formal analysis, investigation, methodology, project administration, resources, supervision, validation, visualization, Writing – original draft, and Writing – review and editing. **Ou Takagi**, **Yutarou Koike**, **Kohei Inaba**, **Mayumi Yamaguchi**, **Yugo Tetsuka**, **Masaru Kuwada**, **Daiki Nemoto**, **Ikuhiro Kobori**, and **Katsutoshi Sugimoto**: writing – review and editing.

## Funding

The authors have nothing to report.

## Ethics Statement

**Institutional review board approval**: Not required for this case report in accordance with institutional policy.

## Consent

Written informed consent for publication was obtained from the patient.

## Conflicts of Interest

The authors declare no conflicts of interest.

## Supporting information




**VIDEO S1**: deo270427‐sup‐0001‐Video.mp4.

**TABLE S1**: Laboratory findings at the referring hospital on presentation.
