# Supplementary material for: Pancreatitis‐Related Encapsulated Fluid Collections Associated With a Pancreatic Duct Stent Retained for 13 Years in Chronic Alcoholic Pancreatitis: A Case Report
Source: DEN Open. 2026 Sep 10;7(1):e70427. doi: 10.1002/deo2.70427 (PMC13562802; doi:10.1002/deo2.70427)
Supplement: Supplementary file 1 [file DEO2-7-e70427-s002.docx]

**Supporting Table S1. Laboratory findings at the referring hospital on presentation**

| **Category** | **Laboratory test** | **Result** | **Reference range** | **Unit** |
| --- | --- | --- | --- | --- |
| Hematology | WBC | 13140 | 4000–8000 | /µL |
|  | Neutrophils | 89.4 | — | % |
|  | Hemoglobin | 10.1 | 14.0–18.0 | g/dL |
|  | MCV | 78.6 | 80.0–99.0 | fL |
|  | Platelets | 354 | 130–350 | ×10³/µL |
| Inflammation | CRP | 21.73 | 0.00–0.30 | mg/dL |
| Hepatobiliary enzymes | Total bilirubin | 0.9 | 0.2–1.0 | mg/dL |
|  | AST | 20 | 12–32 | U/L |
|  | ALT | 21 | 8–36 | U/L |
|  | ALP | 129 | 38–113 | U/L |
|  | γ-GTP | 47 | 9–61 | U/L |
| Nutritional status | Total protein | 6.8 | 6.7–8.3 | g/dL |
|  | Serum albumin | 2.3 | 3.8–5.3 | g/dL |
|  | Cholinesterase | 117 | 220–466 | U/L |
| Pancreatic enzymes | Amylase | 14 | 30–94 | U/L |
| Renal function/electrolytes | BUN | 22.8 | 8.0–20.0 | mg/dL |
|  | Creatinine | 0.51 | 0.30–1.20 | mg/dL |
|  | eGFR | 122.8 | ≥60 | mL/min/1.73 m² |
|  | Sodium | 133 | 134–147 | mEq/L |
|  | Potassium | 4.4 | 3.2–4.8 | mEq/L |
|  | Chloride | 94 | 98–108 | mEq/L |
|  | Calcium | 8.1 | 8.4–11.4 | mg/dL |
| Glucose metabolism | Plasma glucose | 133 | 65–110 | mg/dL |
|  | HbA1c | 7 | 4.6–6.2 | % |

**Abbreviations:** WBC, white blood cell count; MCV, mean corpuscular volume; CRP, C-reactive protein; AST, aspartate aminotransferase; ALT, alanine aminotransferase; ALP, alkaline phosphatase; γ-GTP, gamma-glutamyl transpeptidase; BUN, blood urea nitrogen; eGFR, estimated glomerular filtration rate; HbA1c, glycated hemoglobin.
